# Long-Term Clinical Outcomes in Patients With an Acute ST-Segment-Elevation Myocardial Infarction Stratified by Angiography-Derived Index of Microcirculatory Resistance

**DOI:** 10.3389/fcvm.2021.717114

**Published:** 2021-09-07

**Authors:** Rafail A. Kotronias, Dimitrios Terentes-Printzios, Mayooran Shanmuganathan, Federico Marin, Roberto Scarsini, James Bradley-Watson, Jeremy P. Langrish, Andrew J. Lucking, Robin Choudhury, Rajesh K. Kharbanda, Hector M. Garcia-Garcia, Keith M. Channon, Adrian P. Banning, Giovanni Luigi De Maria

**Affiliations:** ^1^Oxford Heart Centre, NIHR Biomedical Research Centre, Oxford University Hospitals, Oxford, United Kingdom; ^2^OCMR, Radcliffe Department of Medicine, University of Oxford, Oxford, United Kingdom; ^3^Division of Cardiology, Department of Medicine, University of Verona, Verona, Italy; ^4^MedStar Washington Hospital Centre, Washington, DC, United States

**Keywords:** STEMI, NH IMRangio, IMR, prognosis, coronary angiography, coronary physiology

## Abstract

**Aims:** Despite the prognostic value of coronary microvascular dysfunction (CMD) in patients with ST-segment-elevation myocardial infarction (STEMI), its assessment with pressure-wire-based methods remains limited due to cost, technical and procedural complexities. The non-hyperaemic angiography-derived index of microcirculatory resistance (NH IMR_angio_) has been shown to reliably predict microvascular injury in patients with STEMI. We investigated the prognostic potential of NH IMR_angio_ as a pressure-wire and adenosine-free tool.

**Methods and Results:** NH IMR_angio_ was retrospectively derived on the infarct-related artery at completion of primary percutaneous coronary intervention (pPCI) in 262 prospectively recruited STEMI patients. Invasive pressure-wire-based assessment of the index of microcirculatory resistance (IMR) was performed. The combination of all-cause mortality, resuscitated cardiac arrest and new heart failure was the primary endpoint. NH IMR_angio_ showed good diagnostic performance in identifying CMD (IMR > 40U); AUC 0.78 (95%CI: 0.72–0.84, *p* < 0.0001) with an optimal cut-off at 43U. The primary endpoint occurred in 38 (16%) patients at a median follow-up of 4.2 (2.0–6.5) years. On survival analysis, NH IMR_angio_ > 43U (log-rank test, *p* < 0.001) was equivalent to an IMR > 40U(log-rank test, *p* = 0.02) in predicting the primary endpoint (hazard ratio comparison *p* = 0.91). NH IMRangio > 43U was an independent predictor of the primary endpoint (adjusted HR 2.13, 95% CI: 1.01–4.48, *p* = 0.047).

**Conclusion:** NH IMR_angio_ is prognostically equivalent to invasively measured IMR and can be a feasible alternative to IMR for risk stratification in patients presenting with STEMI.

## Introduction

Coronary microvascular dysfunction in patients admitted with ST-segment-elevation myocardial infarction (STEMI) is biologically (1) and prognostically relevant (2, 3). An early assessment of CMD in the catheterisation laboratory is desirable to triage additional therapies and/or plan *ad-hoc* clinical care strategies. This can be achieved by measuring the index of microvascular resistance (IMR) with a conventional pressure-wire and thermodilution technique (4). Our group and others have previously shown the good diagnostic performance of IMR in predicting microvascular injury diagnosed by cardiac magnetic resonance (CMR) imaging (1, 5). Contemporary studies highlight the early- and long-term prognostic implications of an IMR > 40U in patients with STEMI (6, 7). Recently, IMR has also been proposed as a tool to triage novel or additional therapies in STEMI, with promising clinical and research implications (8, 9).

Despite its proposed role as a prognostic biomarker (9) the clinical adoption of IMR remains limited. Additional cost, procedural time, an extra—though small—procedural risk associated with the manipulation of a pressure wire in the infarct related artery (IRA) and patient discomfort due to intravenous adenosine infusion, are amongst some of the barriers to its widespread use. The recent development and application of computational flow dynamics to three-dimensional modeling of the coronary artery represents an excellent opportunity to derive angiographic indices of coronary physiology (such as fractional flow reserve (FFR) or IMR) avoiding the use of a pressure wire (10). Our group has recently described a novel, angiography-derived, pressure-wire free index of microcirculatory resistance (IMR_angio_) (11). This index showed a good diagnostic performance in predicting an invasive IMR > 40U in patients with STEMI. Whilst IMR_angio_ accurately diagnoses CMD in patients with STEMI, it would be desirable to have an adenosine free angiographic index of CMD. It is reported that in most patients with STEMI the vasodilatory capacity of the microvascular coronary bed is blunted and consequently the response of coronary microcirculation to adenosine is often minimal or incomplete. (12, 13). In this regard, we have recently described the non-hyperaemic version of IMR_angio_–NH IMR_angio_, and shown it retains its diagnostic reliability in most patients with STEMI (14). Thus, we hypothesized that NH IMR_angio_ would provide similar prognostic information to invasively-measured IMR.

The aim of this work was to evaluate the prognostic performance of NH IMR_angio_ in patients with STEMI.

## Methods

### Patient Population

Patients with a diagnosis of STEMI presenting between January 2010 and March 2020 with STEMI at the Oxford Heart Centre were enrolled in the prospective OxAMI (Oxford Acute Myocardial Infarction) cohort study (15). The current study is based on prospectively enrolled participants who had pressure-wire based IMR measurements, unless they met any specific exclusion criteria for enrolment (Supplementary Material, Supplementary Figure 1). This analysis includes all participants on whom both invasive IMR measurement and angiography-derived coronary physiology assessment was possible.

Primary percutaneous coronary intervention (pPCI) was performed in standard fashion with the use of adjunctive therapies (manual thrombectomy, glycoprotein IIb/IIIa) and choice of stenting technique at the operator's discretion. ST-segment resolution was calculated using surface electrocardiography acquired before and at 90 min after pPCI as described previously (16), and defined as complete if ≥70%. Post-pPCI left ventricular ejection fraction (LVEF) was derived from echocardiogram performed prior to hospital discharge.

The study protocol was approved by the local ethics committee (10/H0408/24) and conducted in accordance with the Declaration of Helsinki.

### Non-hyperaemic IMR_angio_ Measurement

**Figure 1:**
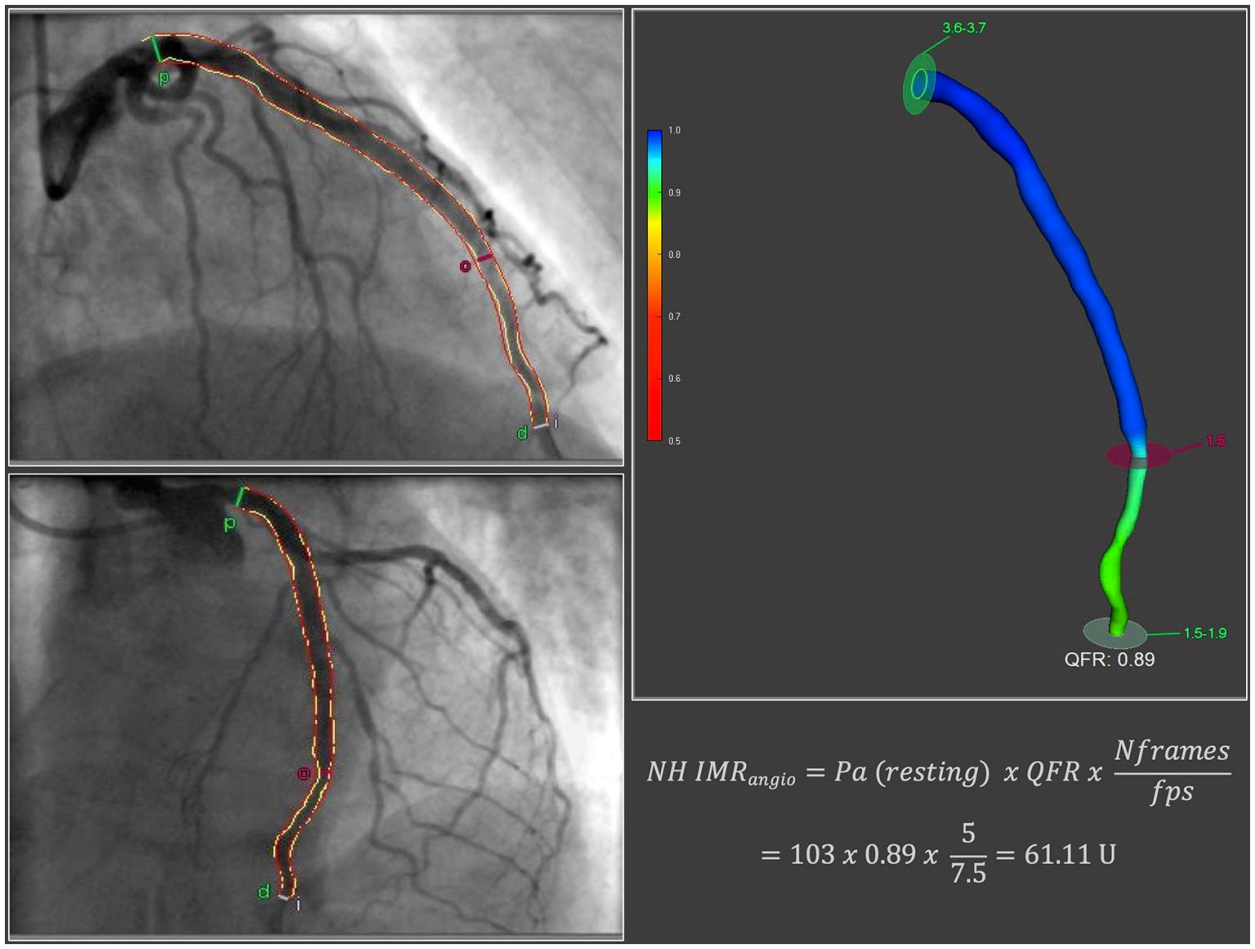
NH IMR_angio_ derivation. fps, frames per second; Nframes, number of angiographic frames from contrast dye to travel from the guiding catheter to a distal reference in resting conditions; NH IMR_angio_, non-hyperaemic IMR_angio_; Pa, post percutaneous coronary intervention mean aortic pressure at resting conditions; QFR, quantitative flow ratio.

The methodology for the derivation of NH IMR_angio_ and its reproducibility have been published previously (14). According to the published methodology, we measured NH IMR_angio_ on the IRA, using two dedicated coronary angiographic projections acquired at the end of the pPCI procedure. Angiographic views were acquired at resting conditions (not during adenosine intravenous infusion). In brief, three-dimensional quantitative coronary angiography and quantitative flow ratio (QFR) analyses were performed using QAngio® XA 3D software (Medis, Leiden, the Netherlands). NH IMR_angio_ was computed using the following formula:

$$\begin{array}{l} NH\;IMRangio=Pa\left(resting\right)\;\times \;QFR\;\times \;\frac{Nframes}{fps} \end{array}$$

where *Pa* was the post PCI mean aortic pressure at resting conditions, *Nframes* was the number of angiographic frames from contrast dye to travel from the guiding catheter to a distal reference (placed at the distal third of the IRA) in resting conditions and fps was the frame-acquisition rate (Figure 1).

All analyses were performed at the OxACT core lab (University of Oxford, Oxford Heart Centre, Oxford, UK) by independent operators blinded to physiology and clinical outcome data.

### Invasive Measurement of Coronary Physiology Indices

Invasive assessment of coronary physiology indices on the IRA was performed with pressure-wire technology (Pressure Wire X, Abbott, California, US or Certus, St. Jude Medical, Minnesota, US) and thermodilution technique at the end of the pPCI as previously reported (Supplementary Material) (15). We routinely collected IMR, coronary flow reserve (CFR) and resistive reserve ratio (RRR) data. Based on established literature, IMR and CFR were dichotomized using thresholds of 40U and 2.0, respectively (5).

### Clinical Follow-Up

The primary clinical outcome of the study was the composite endpoint of all-cause mortality, resuscitated cardiac arrest and new heart failure diagnosis. The secondary clinical outcome of the study was the composite endpoint of cardiac mortality, resuscitated cardiac arrest and new heart failure diagnosis. Heart failure was defined as the development of new heart failure symptomatology and/or prescription of diuretics in conjunction with supporting new non-invasive imaging findings of left ventricular systolic dysfunction (LVEF) < 50% and/or raised levels of brain natriuretic peptide. Outcome data was collected from scheduled study follow-up visits and annual telephone calls and review of electronic case records and contacting the participants' General Practitioners.

### Statistical Analysis

We tested the normality assumption of continuous variables with statistical (Shapiro-Wilk test) and graphical (histogram) means. We expressed continuous variables as mean ± standard deviation or median (25–75 percentile) as appropriate and categorical variables as numbers (percentage). Between-group comparisons for categorical variables were performed using Pearson's χ^2^ or Fisher's exact test as appropriate, while continuous variables were compared using Student's *t*-test, Mann-Whitney U test or Kruskal-Wallis test. Correlations between variables were expressed using Spearman rho coefficients.

We used receiver operator characteristic curve analysis to explore the diagnostic utility of NH IMR_angio_ to predict an IMR > 40U. We then selected the optimal NH IMR_angio_ cut-off value by identifying the value that maximizes the Youden's *J* statistic.

We subsequently performed survival analyses for the primary and secondary endpoints stratified according to (i) NH IMR_angio_, (ii) IMR and (iii) CFR (expressed as dichotomous variables) using Kaplan Meier and Cox proportional hazard regression modeling methods. We compared the hazard ratios of dichotomised NH IMR_angio_, IMR and CFR with paired Student's *t*-tests (17).

To evaluate the prognostic utility of NH IMR_angio_ (as a dichotomous variable) for our primary endpoint we constructed multivariate Cox regression models adjusted for clinical, procedural, angiographic and echocardiographic variables. We measured the goodness of fit using concordance (C-statistic) statistics. By adding NH IMR_angio_ in a baseline multivariate model, we assessed for improvement in model performance by performing a likelihood ratio test.

Statistical analyses were performed with SPSS version 26 (IBM Inc. New York USA) and R studio version 1.3 (*survival, survminer, forestplot* and *survcomp* packages). All tests were two-sided and α was set at 0.05.

## Results

### Study Population

A total of 262 patients with both post pPCI invasive IMR and NH IMR_angio_ assessment were included in the current analysis. Complete follow-up data was available for 241 participants (92%) with a median follow-up of 4.2 (2.0–6.5) years. Baseline clinical, procedural, imaging and post pPCI coronary physiology data are reported in Tables 1, 2. The median IMR and NH IMR_angio_ values at the end of the procedure were 33 (20–55) and 43 (30–59) units respectively.

**Table 1:** Clinical, procedural and echocardiographic characteristics.

	**All**
**Total number**	**262**
**Clinical**	
Age, years	62 ± 11
Male gender, *n* (%)	215 (82)
Hypertension, *n* (%)	119 (46)
Hypercholesterolemia, *n* (%)	101 (39)
Diabetes, *n* (%)	41 (16)
Smoker, *n* (%)	110 (42)
Previous cardiology history, *n* (%)	37 (14)
Family history of IHD, *n* (%)	101 (39)
**Procedural**	
Ischemic time (minutes)	180 (122, 317)
*Target vessel*	
LAD, *n* (%)	119 (45)
LCX, *n* (%)	25 (10)
RCA, *n* (%)	109 (42)
Other, *n* (%)	9 (3)
TIMI flow—pre-PCI	
0	197 (75)
1	22 (8)
2	30 (12)
3	13 (5)
TIMI flow—post-PCI	
0	0 (0)
1	3 (1)
2	33 (13)
3	226 (86)
Myocardial Blush Grade	
0	21 (8)
1	46 (18)
2	106 (40)
3	89 (34)
**Complete ST segment resolution**, ***n*****(%)**	151 (73)
**Discharge echocardiography LVEF (%)**	50 (45, 56)

*IHD, ischaemic heart disease; LAD, left anterior descending artery; LCx, left circumflex artery; LVEF, left ventricle ejection fraction; PCI, percutaneous coronary intervention; RCA, right coronary artery; TIMI, the Thrombolysis in Myocardial Infarction*.

**Table 2:** Post PCI pressure-wire- and angiography-derived coronary physiology indices.

	**All**
**Total number**	**262**
**Pressure-wire-derived**	
Resting Pa, mmHg	92 ± 18
Resting transit time, s	0.69 (0.48, 1.13)
Hyperaemic Pa, mmHg	83 ± 16
Hyperaemic Pd, mmHg	76 (67, 87)
Hyperaemic transit time, s	0.43 (0.28, 0.78)
FFR	0.94 (0.90, 0.98)
IMR	33 (20, 55)
CFR	1.5 (1.1, 2)
RRR	1.7 (1.3, 2.3)
**Angiography-derived**	
NH IMR_angio_	43 (30, 59)
Fixed flow QFR	0.95 (0.90, 0.98)
Contrast QFR	0.96 (0.90, 0.99)

*CFR, coronary flow reserve; FFR, fractional flow reserve; IMR, index of microvascular resistance; NH IMR_angio_, non-hyperaemic IMR_angio_; Pa, aortic pressure; Pd, distal pressure; PCI, percutaneous coronary intervention; QFR, quantitative flow ratio; RRR, resistive reserve ratio*.

### Diagnostic Performance of NH IMR_angio_

NH IMR_angio_ was significantly correlated with IMR (rho = 0.50, *p* < 0.0001) and predicted an IMR > 40U with an AUC of 0.78 (95% CI: 0.72–0.84, *p* < 0.0001) (Supplementary Figure 2). The optimal NH IMR_angio_ cut-off to predict an IMR > 40U was 43U (Sensitivity: 77%, Specificity: 67%, Diagnostic accuracy: 70%) coinciding with the median NH IMR_angio_ value of 43U observed in the overall study cohort. Patients with a high NH IMR_angio_ (>43U) were characterized by longer ischaemic times, worse post pPCI TIMI flow grade, lower occurrence of complete ST-segment resolution, and lower left ventricular ejection fraction at discharge than patients with a low NH IMR_angio_ (Supplementary Table 1). A high NH IMR_angio_ (>43U) was associated with a significantly higher degree of invasively assessed microvascular dysfunction (expressed by either IMR, CFR or RRR) than a low NH IMR_angio_ (Supplementary Table 2).

### Prognostic Value of NH IMR_angio_

At 7 years of follow-up, the primary and secondary endpoints occurred in 38 (16%) and 30 (12%) participants respectively. All-cause death occurred in 13 (5%) participants (4 cardiac, 1 sepsis-related and 6 neoplasm-related deaths), two (1%) had a resuscitated cardiac arrest and 28 (12%) had a new diagnosis of heart failure. Cox regression analyses showed that a post pPCI NH IMR_angio_ >43U was significantly associated with a higher risk of both the primary and secondary endpoints, (HR 3.43, 95% CI: 1.67–7.07, *p* = 0.001 and HR 3.32, 95% CI: 1.48–7.47, *p* = 0.004, respectively). A post pPCI IMR >40U was also significantly associated with a higher risk of the primary and secondary endpoints (HR 2.07, 95% CI: 1.09–3.92, *p* < 0.03 and HR 2.17, 95% CI 1.06–4.48, *p* < 0.04, respectively). The comparison of the hazard ratio estimates of NH IMR_angio_ > 43U and IMR > 40U did not yield a statistically significant difference for either endpoint (*p* = 0.91 and *p* = 0.85).

In an exploratory analysis, a CFR ≤ 2.0 was significantly associated with a higher risk of the primary and secondary endpoints (HR 3.82, 95% CI 1.17–12.43, *p* < 0.03 and HR 4.56, 95% CI 1.08–19.18, *p* < 0.04, respectively). Pairwise comparisons of CFR ≤ 2.0 with IMR > 40U and NH IMR_angio_ > 43U hazard ratios were not statistically significant.

**Figure 2:**
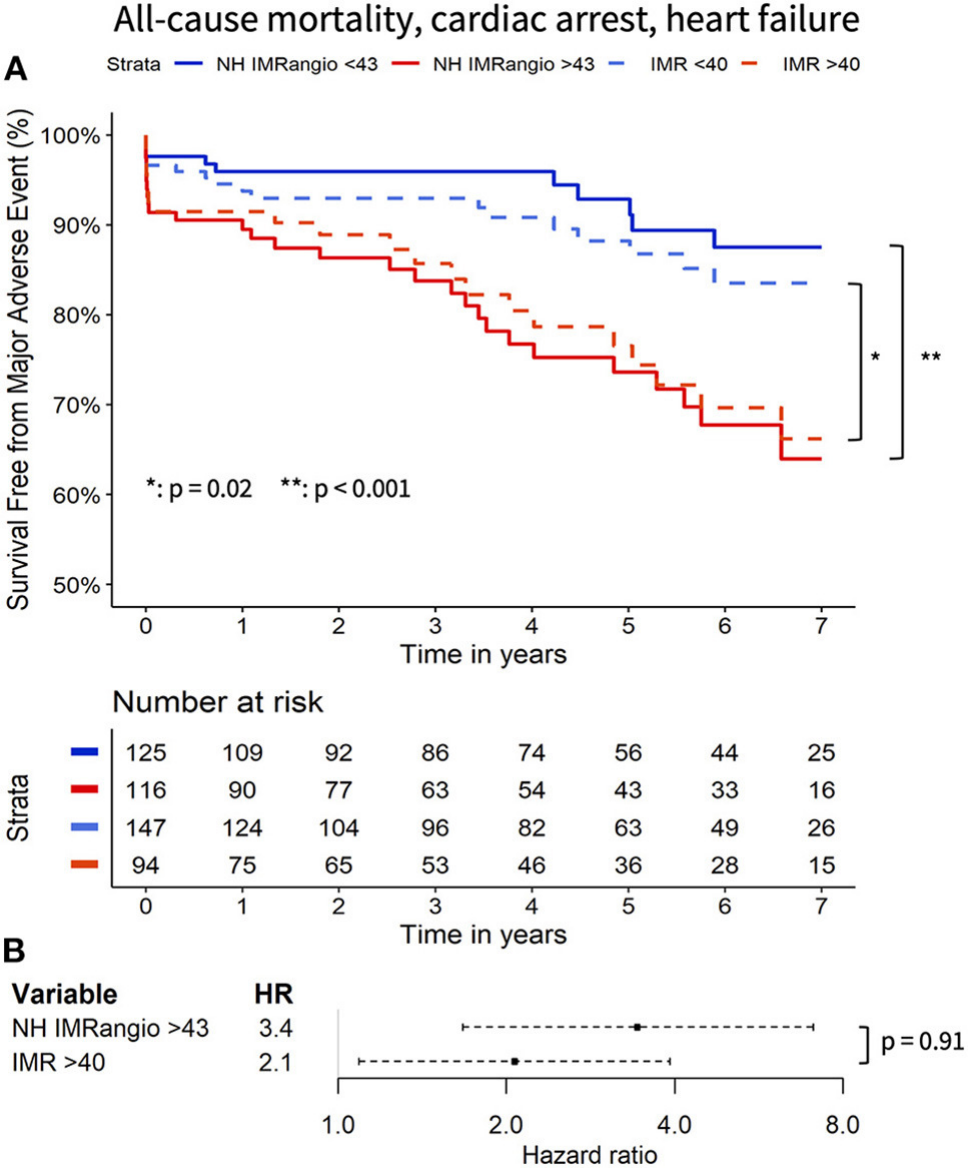
Kaplan Meier curves of freedom from all-cause mortality, resuscitated cardiac arrest, new heart failure diagnosis with high vs. low (i) NH IMR_angio_ and (ii) IMR **(A)**. Forrest plot displaying the hazard ratio of high (i) NH IMR_angio_ and (ii) IMR **(B)**. HR, hazard ratio; IMR, index of microcirculatory resistance; NH IMR_angio_, non-hyperaemic IMR_angio_.

**Figure 3:**
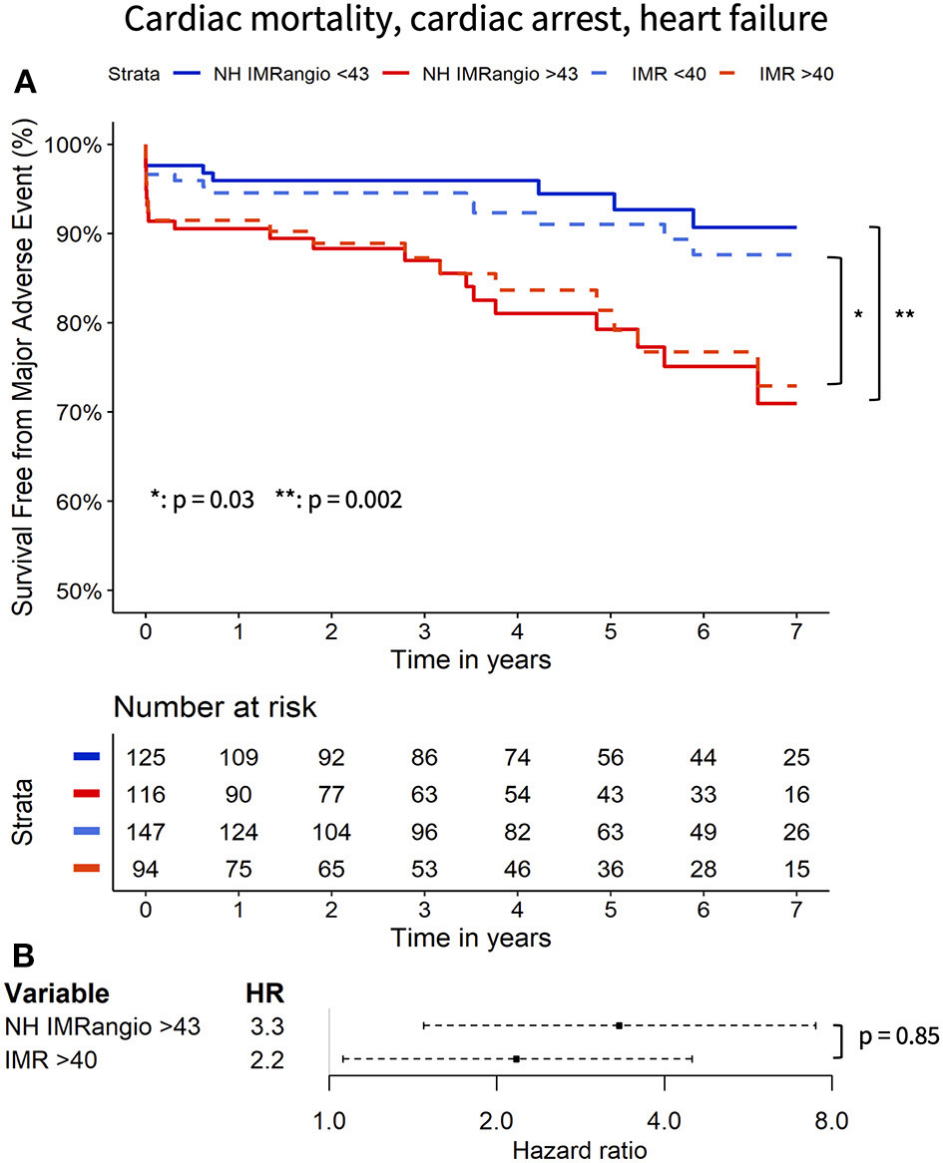
Kaplan Meier curves of freedom from cardiac mortality, resuscitated cardiac arrest, new heart failure diagnosis with high vs. low (i) NH IMR_angio_ and (ii) IMR **(A)**. Forrest plot displaying the hazard ratio of high (i) NH IMR_angio_ and (ii) IMR **(B)**. HR, hazard ratio; IMR, index of microcirculatory resistance; NH IMR_angio_, non-hyperaemic IMR_angio_.

**Figure 4:**
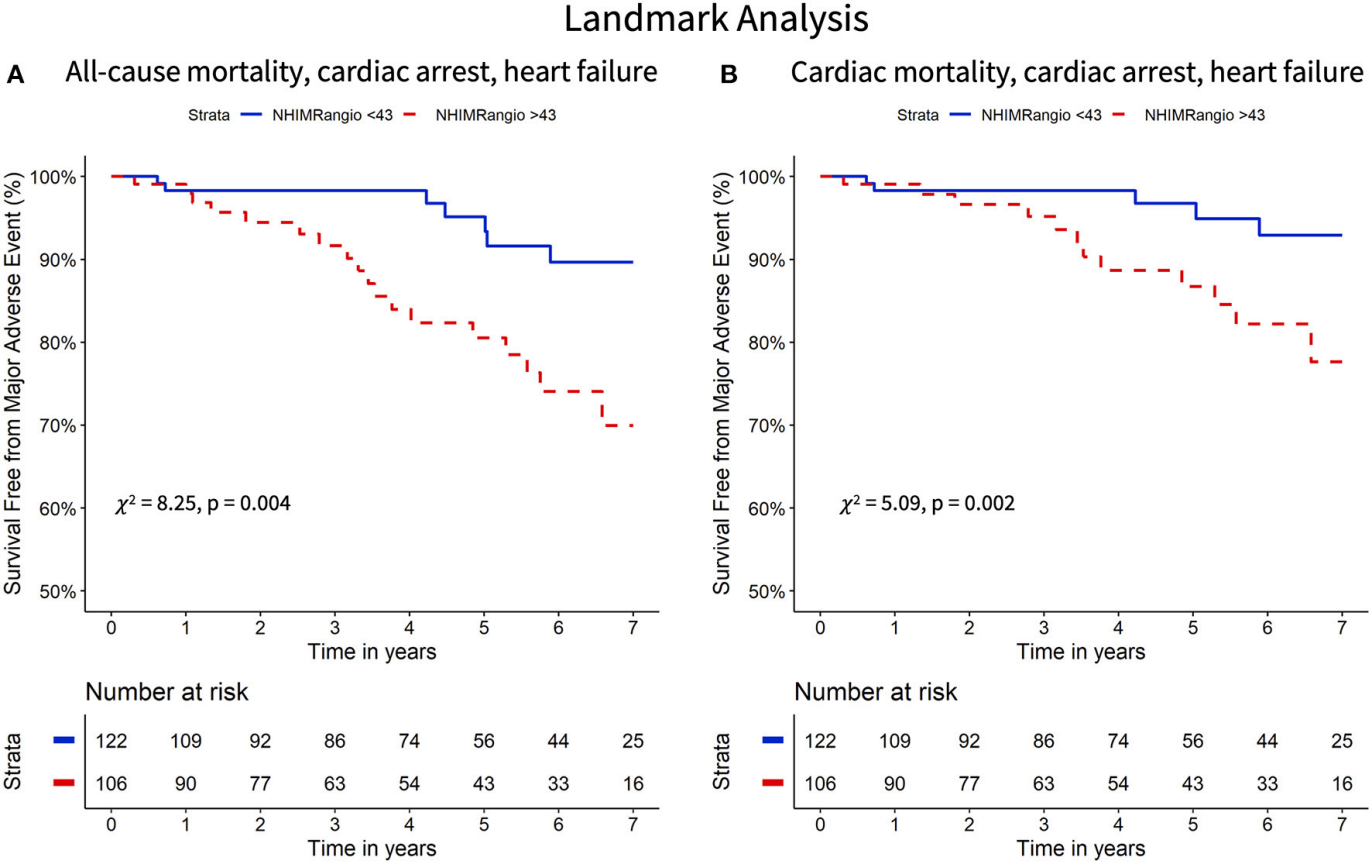
Landmark analysis (30 days onward) Kaplan Meier curves of freedom from all-cause mortality, resuscitated cardiac arrest, new heart failure diagnosis **(A)** and cardiac mortality, resuscitated cardiac arrest and new heart failure diagnosis **(B)** stratified according to high versus low NH IMR_angio_. NH IMR_angio_, non-hyperaemic IMR_angio_.

The Kaplan Meier curves displaying the relationship between survival-free from the primary and secondary endpoints for participants with high vs. low NH IMR_angio_ and IMR have a similar profile (Figures 2, 3 respectively). The analysis of participants dichotomised according to low vs. high CFR yield similar Kaplan Meier survival curve profiles to the profiles observed when participants were dichotomised according to IMR and NH IMR_angio_ for both endpoints (Supplementary Figures 3, 4). In a landmark analysis excluding participants who experienced a primary or secondary endpoint event at 30 days, survival free from a primary and secondary endpoint was significantly lower in participants with an NH IMR_angio_ > 43U (Figure 4). In a further sub-analysis excluding patients with post pPCI TIMI Flow grade <3, NH IMR_angio_ > 43U was significantly associated with a higher risk of the primary endpoint (Supplementary Figure 5).

**Figure 5:**
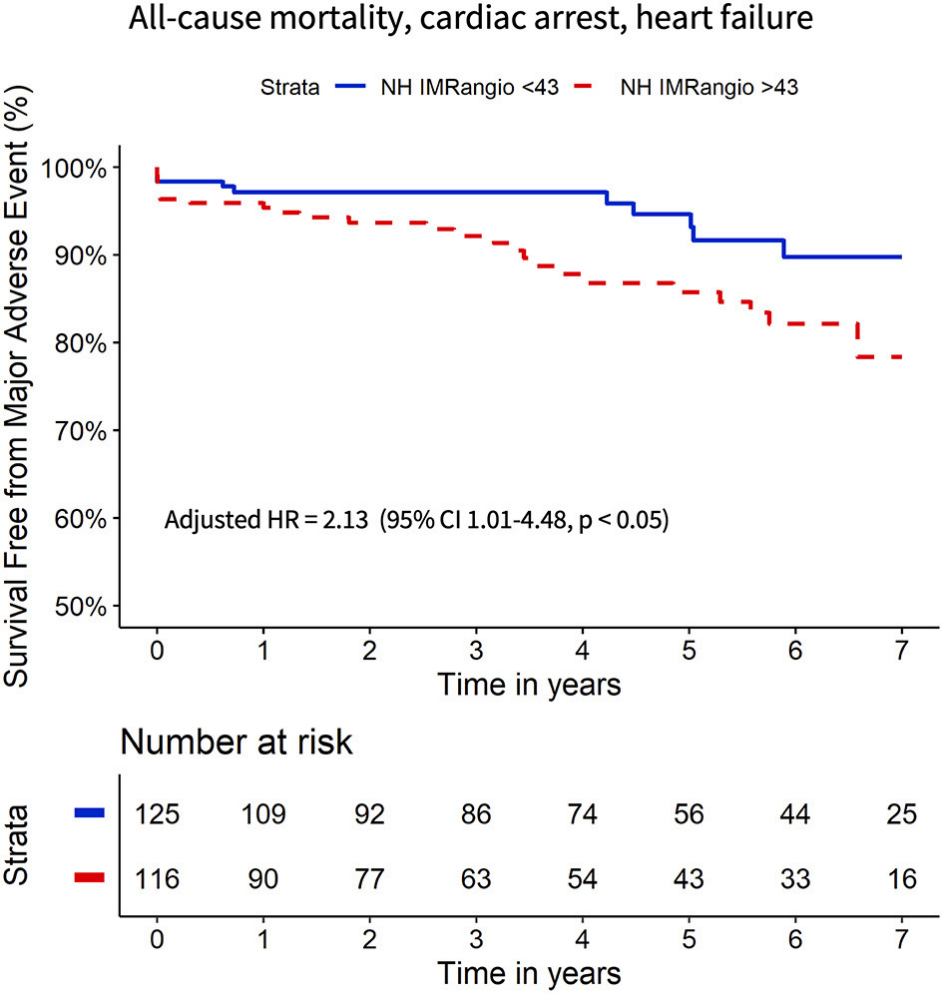
Kaplan Meier curves of freedom from all-cause mortality, resuscitated cardiac arrest, new heart failure diagnosis with high vs. low NH IMR_angio_ adjusted for age, ischaemic time, LVEF%. HR, hazard ratio; CI, confidence interval; LVEF, left ventricle ejection fraction; NH IMR_angio_, non-hyperaemic IMR_angio_.

To further evaluate the prognostic utility of NH IMR_angio_ >43U a multivariate Cox regression analysis was performed. The univariate and multivariate predictors of the primary endpoint are listed in Table 3. Post pPCI TIMI flow grade <3, myocardial blush grade <3 and incomplete ST-segment resolution were not significant predictors (Table 3). NH IMR_angio_ >43U was an independent predictor of the primary endpoint (adjusted HR 2.13, 95% CI: 1.01–4.48, *p* = 0.047) alongside age, ischaemic time, and discharge LVEF (Figure 5). NH IMRangio >43U was an independent predictor of long-term outcome even when patients with post pPCI TIMI flow <3 were excluded from the analysis (adjusted HR 2.82, 95% CI: 1.28–6.19, *p* = 0.01). The addition of NH IMR_angio_ >43U as a variable to a cox regression model including age, ischaemic time and discharge LVEF% yielded a good model (C-statistic 0.82, χ^2^: 67) with a significant improvement in predictive performance (χ^2^ difference: 4.30, *p* = 0.04).

**Table 3:** Univariate and multivariate analysis results for predictors of all-cause mortality, resuscitated cardiac arrest, new heart failure.

**Univariate predictors**	**Hazard ratio**	**95% CI**	**p-value**
Age (per 1 year increase)	1.07	1.03–1.10	** <0.01**
Male gender	2.02	1.00–4.07	0.05
LAD as IRA	2.09	1.09–4.00	**0.03**
Ischaemic time (per 1 min delay)	1.00	1.00–1.00	**0.04**
Incomplete ST segment resolution	2.20	0.82–5.91	0.12
TIMI Flow Grade <3	1.32	0.58–3.00	0.51
Myocardial Blush Grade <3	1.61	0.76–3.39	0.22
IMR > 40	2.07	1.09–3.92	** <0.03**
CFR **≤** 2.0	3.82	1.17–12.43	** <0.03**
Discharge echocardiography LVEF (per % increase)	0.91	0.89–0.94	** <0.01**
NH IMR_angio_ > 43U	3.43	1.67–7.07	** <0.01**
**Multivariate predictors**			
Age (per 1 year increase)	1.07	1.03–1.11	** <0.01**
Ischaemic time (per 1 min delay)	1.00	1.00–1.00	**0.03**
Discharge echocardiography LVEF (per % increase)	0.92	0.90–0.95	** <0.01**
NH IMR_angio_ > 43U	2.13	1.01–4.48	** <0.05**

*CI, confidence interval; CFR, coronary flow reserve; IMR, index of microvascular resistance; IRA, infarct related artery; LAD, left anterior descending artery; LVEF, left ventricle ejection fraction; NH IMR_angio_, non-hyperaemic IMR_angio_; TIMI, Thrombolysis in Myocardial Infarction. Values in bold denote a statistically significant result (p-value <0.05)*.

## Discussion

This study extends our preliminary findings (14) and explores the prognostic value of a dedicated pressure-wire and adenosine-free angiographic index (NH IMR_angio_) for the assessment of CMD in patients with STEMI.

Coronary microvascular dysfunction in STEMI is prognostically important (2, 7) due to the resulting poor structural and functional myocardial recovery (1, 18). Physiological measurements derived from invasive assessment of coronary microvascular function at the time of pPCI (e.g. IMR) provide a grading of the severity of CMD and correlate well with CMR-defined structural microvascular injury (1, 4). However, the routine clinical adoption of IMR remains limited due to a number of factors including additional cost and procedural time. These constraints can be overcome with angiography-derived, pressure wire-free indices of coronary physiology indices. Our recent validation of the non-hyperaemic angiography-derived index of microcirculatory resistance (NH IMR_angio_) against pressure-wire-based IMR represented a preliminary dataset (14).

### Diagnostic Performance of NH IMR_angio_

In the current analysis, we expand our preliminary findings to a larger cohort and show that the non-hyperaemic version of IMR_angio_–NH IMR_angio_ reliably predicts CMD as defined by an IMR >40U in the IRA of patients with STEMI. Using a non-hyperaemic index to evaluate CMD in the IRA of patients with STEMI could have a certain value, as the response of coronary microcirculation to a vasodilatory agent is highly likely to be blunted and compromised as a reflection of distal embolisation and ischaemia-reperfusion injury (5, 13). As a confirmation of this hypothesis, in our cohort, the median value of RRR—a dedicated index to express the vasodilatory capacity of the coronary microcirculation—was 1.7. This value is suggestive of a depressed coronary microvascular vasodilatory capacity in this cohort of patients with STEMI, a finding consistent with previous reports (12, 13). This depressed vasodilatory capacity can explain why, particularly in patients with STEMI, a non-hyperaemic index such as NH IMR_angio_ retains some diagnostic accuracy in identifying microvascular injury (14).

### Prognostic Value of NH IMR_angio_

The main finding of the current study is the observation that an NH IMR_angio_ >43U, measured in the IRA of patients with STEMI at the end of pPCI, is equivalent to an IMR >40U in predicting long-term adverse events. The survival curves of patients stratified according to low or high values of IMR or NH IMR_angio_ present a similar profile, while the stratification is prognostically significant for both indices. The hazard ratios of a high IMR (>40U) or NH IMR_angio_ (>43U) are not significantly different, further supporting the prognostic equivalence of the two indices. The prognostic equivalence is also maintained when analysing a stricter cardiac endpoint excluding non-cardiac mortality. These findings are consistent with the results and effect estimates reported in the previous work by Fearon et al. on the prognostic role of invasive and pressure-wire based IMR in patients with STEMI (7).

Kaplan-Meier survival curves separate early on, suggesting a prognostic role of NH IMR_angio_ for early cardiac complications; a finding corroborated by previous IMR based work (6). Since this early separation could influence our analysis, we conducted a landmark analysis from 30 day onwards. An NH IMR_angio_ >43U retained its significance in this landmark analysis suggesting that the long-term prognostic performance is not exclusively driven by early events. This may be explained by the significant contribution of new heart failure diagnoses to our combined study endpoints. In our previous work, we have already shown that non-hyperaemic IMR_angio_ is significantly elevated in patients with clinically significant microvascular obstruction assessed by cardiac magnetic resonance (14). This provides a further biologically plausible explanation for the prognostic significance of NH IMR_angio_ we report herein (14).

Finally, this study suggests that an NH IMR_angio_ >43U is an independent predictor of adverse events, with an associated two-fold increased risk of a poor clinical outcome at 7 years follow-up. Our findings resonate with previously published findings of the independent prognostic value of an IMR > 40U in predicting long-term outcomes in patients with STEMI (5, 7). Specifically, adding NH IMR_angio_ into a model with other clinically relevant and universally available variables incrementally improved the predictive performance of the model itself, supporting the independent and incremental prognostic significance of NH IMR_angio_ as a novel alternative tool for risk stratification.

### Limitations

Firstly, we acknowledge that the relatively small sample size, low number of cardiac events and single-centre origin of our study is amongst the limitations of our work. For this reason, further testing in larger and external cohorts is needed to corroborate and to increase the precision of the findings. We also recognize that our cohort study might have been subject to selection bias due to the exclusive inclusion of patients in whom invasive coronary physiology measurements were performed. This might have led to the unintentional inclusion of a relatively intermediate-low risk cohort of patients with STEMI, as reflected by the relative low rate of adverse events at follow up. However, on a practical level it must also be considered that the real unmet need is to improve risk-stratification in patients at intermediate risk of adverse events. Patients presenting with high-risk features (multiple comorbidities, haemodynamic instability, complex coronary anatomy) have already “declared” their risk category, whilst it is the majority of “intermediate risk” patients (like the patients included in our analysis) that may benefit the most from personalized and stratified medicine approaches (19).

In addition, we recognize the limitations arising from the use of a non-hyperaemic index to study coronary microvascular function. A future large-scale, prospective study assessing the prognostic value of the hyperaemic angiography-derived index is indeed needed, and it could abate the limitations of the current analysis focused on a non-hyperaemic version of the index. Nonetheless, it must be acknowledged that in the current study, NH IMR_angio_ resulted prognostically equivalent to invasive IMR. Whilst this observation is evident in STEMI patients, we do not anticipate that a non-hyperaemic index will work as well in patients with coronary syndromes characterized by a lesser degree of CMD, as also already recently reported (14). Finally, a limitation of this work is the off-line evaluation of NH IMR_angio_ and our ability to perform the analysis in 71% of patients. Even though our tool should be formally evaluated in a “real-time” setting, there is little doubt about its suitability as a real-time catheterisation laboratory tool. A large body of evidence suggests that real-time measurement of QFR is not only feasible but significantly quicker than pressure-wire based coronary physiology evaluation (20). Indeed, we expect that dedicated acquisitions for real-time assessment of NH IMR_angio_ in a prospective study would enable analysis in >95% of cases.

## Conclusion

In an analysis of 262 patients with STEMI, NH IMR_angio_ is reliable at predicting CMD defined as an IMR >40U. An NH IMR_angio_ of >43U was an independent multivariate associate of long-term all-cause mortality, resuscitated cardiac arrest and new heart failure diagnosis. Since the prognostic profile of NH IMR_angio_ was equivalent to that of IMR, it can be a feasible alternative to IMR for catheterisation laboratory assessment of CMD and long-term risk stratification.

## Data Availability Statement

The datasets presented in this article are not readily available because a patent based on some of the data included in this article has been filed. Requests to access the datasets should be directed to Giovanni Luigi De Maria, GiovanniLuigi.Demaria@ouh.nhs.uk.

## Ethics Statement

The studies involving human participants were reviewed and approved by the local ethics committee (10/H0408/24). The patients/participants provided their written informed consent to participate in this study.

## Author Contributions

RAK: methodology, investigation, formal analysis, writing—original draft, visualisation, writing—review and editing, project administration, and funding acquisition. DT-P, FM, RS, JB-W, JL, AL, RC, and RAK: investigation and writing—review and editing. MS: investigation, writing—review and editing, and funding acquisition. OxAMI Study Investigators: investigation, methodology, resources, project administration, and funding acquisition. HG-G: conceptualisation, methodology, and writing—review and editing. KC: conceptualisation, methodology, investigation, resources, writing—review and editing, project administration, and funding acquisition. AB: conceptualisation, methodology, investigation, resources, writing—review and editing, and funding acquisition. GD: conceptualisation, methodology, investigation, resources, writing—original draft, writing—review and editing, supervision, project administration, and funding acquisition. All authors contributed to the article and approved the submitted version.

## Funding

This study was Supported by British Heart Foundation (BHF; grant CH/16/1/32013), BHF Centre of Research Excellence, Oxford (RG/13/1/30181), Oxfordshire Health Services Research Committee and the National Institute for Health Research (NIHR) Oxford Biomedical Research Centre. RAK, Academic Clinical Fellow is funded by Health Education England (HEE) /National Institute for Health Research (NIHR) for this research project. MS is in receipt of the Alison Brading Memorial Scholarship in Medical Science, Lady Margaret Hall, University of Oxford.

## Author Disclaimer

The views expressed in this publication are those of the authors and not necessarily those of the NIHR, NHS or the UK Department of Health and Social Care.

## Conflict of Interest

GD reports grants from Miracor Medical SA, outside the submitted work. In addition, GD has a patent PCT/US20/55240 pending. AB reports grants from Boston Scientific, personal fees from Boston Scientific, personal fees from Abbott, personal fees from Medtronic, personal fees from Phillips, outside the submitted work. HG-G reports institutional research grants from Medtronic, Boston Scientific, Abbott, Biotronik, Neovasc, Corflow, Shockwave, Chiesi, outside the submitted work. The remaining authors declare that the research was conducted in the absence of any commercial or financial relationships that could be construed as a potential conflict of interest.

## Publisher's Note

All claims expressed in this article are solely those of the authors and do not necessarily represent those of their affiliated organizations, or those of the publisher, the editors and the reviewers. Any product that may be evaluated in this article, or claim that may be made by its manufacturer, is not guaranteed or endorsed by the publisher.

## Abbreviations

CFR: coronary flow reserve
CMD: coronary microvascular dysfunction
IMR: index of microcirculatory resistance
LVEF: left ventricle ejection fraction
NH IMR_angio_: non-hyperaemic IMR_angio_
pPCI: primary percutaneous coronary intervention
QFR: quantitative flow ratio
RRR: resistive reserve ratio
STEMI: ST-segment elevation myocardial infarction.
