# Supplementary material for: Long-Term Clinical Outcomes in Patients With an Acute ST-Segment-Elevation Myocardial Infarction Stratified by Angiography-Derived Index of Microcirculatory Resistance
Source: Front Cardiovasc Med. 2021 Sep 7;8:717114. doi: 10.3389/fcvm.2021.717114 (PMC8452918; doi:10.3389/fcvm.2021.717114)
Supplement: Supplementary file 1 [file Data_Sheet_1.pdf]

Supplementary Methods

STEMI diagnosis

STEMI was diagnosed in the presence of chest pain lasting for at least 30 minutes accompanied by ST segment elevation (>2 mm) in at least 2 anatomically contiguous leads.

Pressure-wire based measurements

Pressure-wire-based coronary physiology was not performed if any of the following exclusion criteria were met: i) haemodynamic instability, ii) history of coronary artery bypass grafting, iii) severe chronic renal failure, iv) angiographic evidence of severe left main disease or complex coronary anatomy (tortuous IRA, presence of a chronic total occlusion), and v) pPCI performed with plain old balloon angioplasty.

Briefly, measurements were taken after intracoronary injection of 250μg of isosorbide dinitrate. Transit time was calculated as the average of transit time measurements during three separate injections of 3 ml of 0.9% room temperature saline at resting and hyperaemic conditions. Hyperaemia was induced by intravenous adenosine infusion at a rate of 140μg/kg/min. IMR was defined as the product of mean distal coronary pressure and mean transit time during hyperaemia.[4] Coronary flow reserve (CFR) was expressed as the ratio of resting to hyperaemic mean transit times.[4] Resistive reserve ratio (RRR) was defined as the ratio of resting to hyperaemic coronary microcirculatory resistance.[11]

TIMI Flow grade

The Thrombolysis in Myocardial Infarction (TIMI) trial grading system was used as a semiquantitative angiographic tool to assess coronary flow before and at completion of pPCI on the IRA

Statistical Methods

Missingness for ST segment resolution and CFR ≤2.0 was addressed by creating a third group for the variable (yes/no/unknown) whilst median imputation was utilised for discharge echocardiography LVEF. Proportional hazard assumptions were graphically and statistically assessed. Explanatory variables with a p-value of <0.1 at univariate analysis were entered in the model using a conditional backward stepwise method.

Supplementary Table

Table S1. Clinical, Procedural & Echocardiographic Characteristics according to low versus high NH IMR_{angio}

	NH IMR_{angio} ≤43U	NH IMR_{angio} >43U	p value
**Total Number**	**136**	**126**	
**Clinical**			
Age, years	60 ± 11	64 ± 11	**<0.01**
Male gender, n (%)	118 (87)	97 (77)	**0.04**
Hypertension, n (%)	56 (42)	63 (50)	0.17
Hypercholesterolemia, n (%)	50 (37)	51 (41)	0.54
Diabetes, n (%)	23 (17)	18 (14)	0.56
Smoker, n (%)	66 (49)	44 (35)	**0.03**
Previous cardiology history, n (%)	17 (13)	20 (16)	0.45
Family history of IHD, n (%)	55 (40)	46 (37)	0.51
**Procedural**			
Ischemic time, minutes	158 (113, 293)	201 (137, 337)	**0.02**
Target vessel			
LAD, n(%)	55 (41)	64 (51)	
LCX, n(%)	12 (9)	13 (10)	0.37
RCA, n(%)	63 (46)	46 (37)	
Other, n(%)	6 (4)	3 (2)	

TIMI flow – pre-PCI			
0	103 (76)	94 (75)	
1	11 (8)	11 (9)	0.84
2	14 (10)	16 (13)	
3	8 (6)	5 (4)	
TIMI flow – post-PCI			
0	0 (0)	0 (0)	
1	0 (0)	3 (2)	**<0.01**
2	8 (6)	25 (20)	
3	128 (94)	98 (78)	
**Complete ST segment resolution, n(%)**	87 (82%)	64 (64%)	**<0.01**
**Discharge echocardiography LVEF, %**	53 (47, 56)	49 (43, 55)	**0.03**

IHD: ischaemic heart disease; LAD: left anterior descending artery; LCx: left circumflex artery; LVEF: left ventricle ejection fraction; MVO: microvascular obstruction; NH IMR_{angio}: non-hyperaemic IMR_{angio}; PCI: percutaneous coronary intervention; RCA: right coronary artery; TIMI: the Thrombolysis in Myocardial Infarction. Values in bold denote a statistically significant result (p-value <0.05).

Table S2. Post PCI invasive and angiography-derived coronary physiology indices according to low versus high NH IMR_{angio}.

	**NH IMR_{angio} ≤43U**	**NH IMR_{angio} >43U**	**p-value**
**Total Number**	**136**	**126**	
**Pressure-wire-derived**			
Resting Pa, mmHg	88 ± 16	97 ± 18	**<0.01**
Resting transit time, s	0.59 (0.38, 0.83)	0.88 (0.59, 1.40)	**<0.01**
Hyperaemic Pa, mmHg	80 (70, 89)	85 (74, 95)	**<0.01**
Hyperaemic Pd, mmHg	72 ± 14	82 ± 16	**<0.01**
Hyperaemic transit time, s	0.33 (0.24, 0.55)	0.59 (0.36, 1.03)	**<0.01**
FFR	0.92 (0.88, 0.97)	0.95 (0.90, 0.99)	**<0.01**
IMR	25 (17, 35)	46 (29, 85)	**<0.01**
CFR	1.6 (1.2, 2.1)	1.4 (1.1, 1.9)	**<0.05**
RRR	1.8 (1.3, 2.5)	1.6 (1.2, 2.2)	**0.02**
**Angiography-derived**			
NH IMR_{angio}	31 (22, 37)	60 (50, 77)	**<0.01**
Fixed flow QFR	0.95 (0.90, 0.98)	0.96 (0.90, 0.99)	0.65
Contrast QFR	0.95 (0.89, 0.98)	0.97 (0.91, 0.99)	**<0.04**

CFR: coronary flow reserve; FFR: fractional flow reserve; IMR: index of microvascular resistance; NH IMR_{angio}: non-hyperaemic IMR_{angio}; Pa: aortic pressure; Pd: distal pressure; PCI: percutaneous coronary intervention; QFR: quantitative flow ratio; RRR: resistive reserve ratio. Values in bold denote a statistically significant result (p-value <0.05).

Supplementary Figures

Figure S1

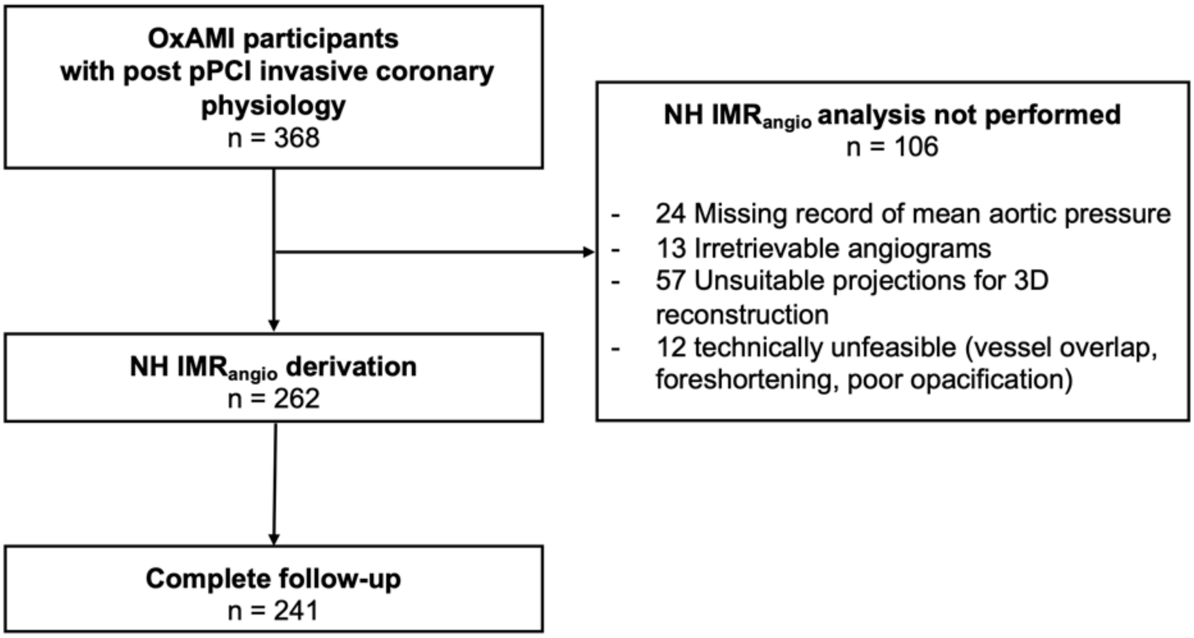

Figure S1: Patient flow diagram

NH IMR_{angio}: non-hyperaemic IMR_{angio}.; pPCI: primary percutaneous coronary intervention; 3D: three dimensional.

Figure S2

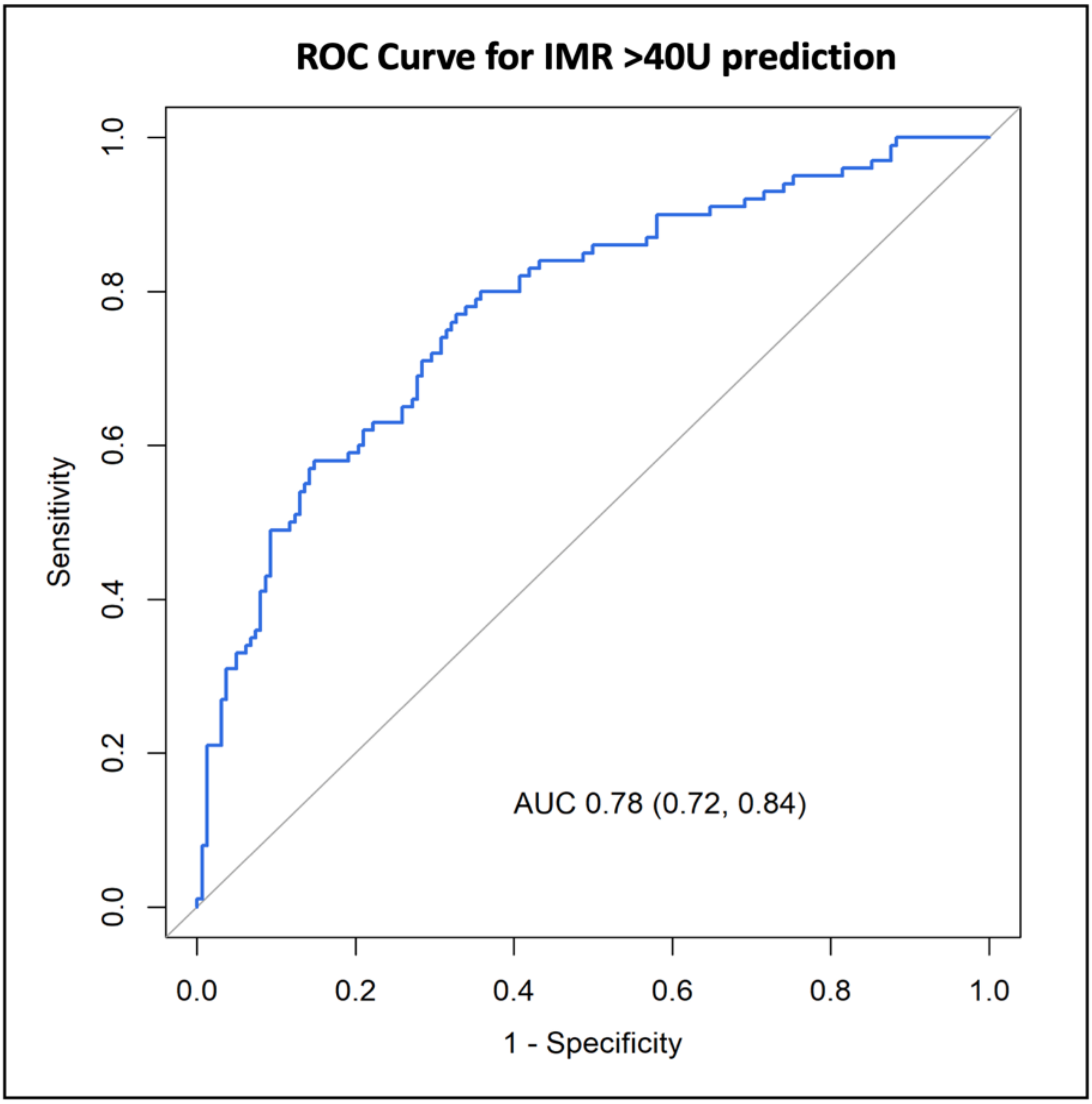

Figure S2: Diagnostic utility of NH IMR_{angio} in identifying an IMR >40U.

AUC: area under the curve; IMR: index of microcirculatory resistance; NH IMR_{angio}*: non-hyperaemic* IMR_{angio}*; ROC: receiver operating characteristic.*

Figure S3

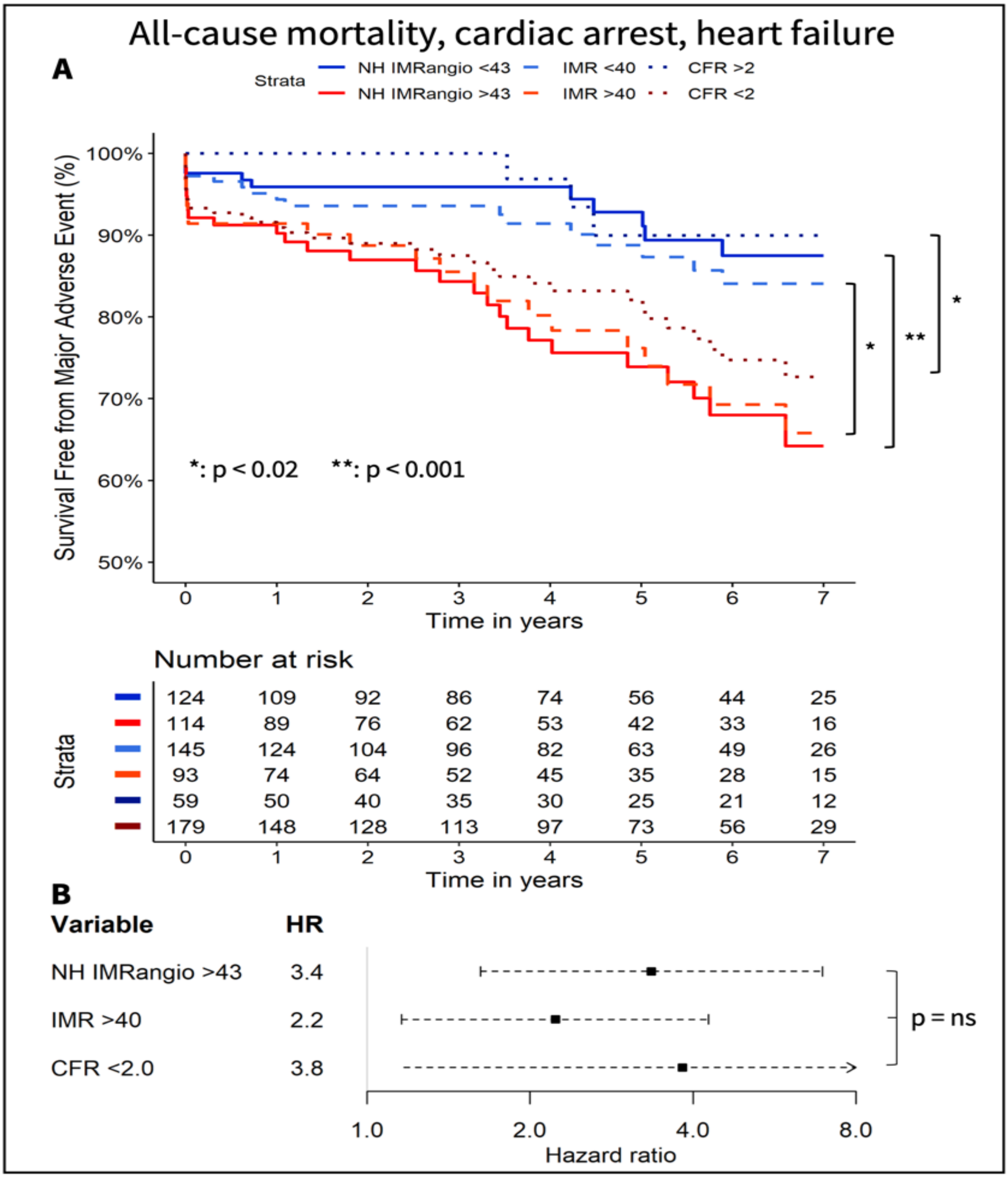

Figure S3: Kaplan Meier Curves of freedom from all-cause mortality, resuscitated cardiac arrest, new heart failure diagnosis with high versus low i) NH IMR_{angio} and ii) IMR **(A)**. Forrest plot displaying the hazard ratio of high i) NH IMR_{angio} and ii) IMR **(B)**.

CFR: coronary flow reserve; HR: hazard ratio; IMR: index of microcirculatory resistance; NH IMR_{angio}: non-hyperaemic IMR_{angio}; ns= not significant.

Figure S4

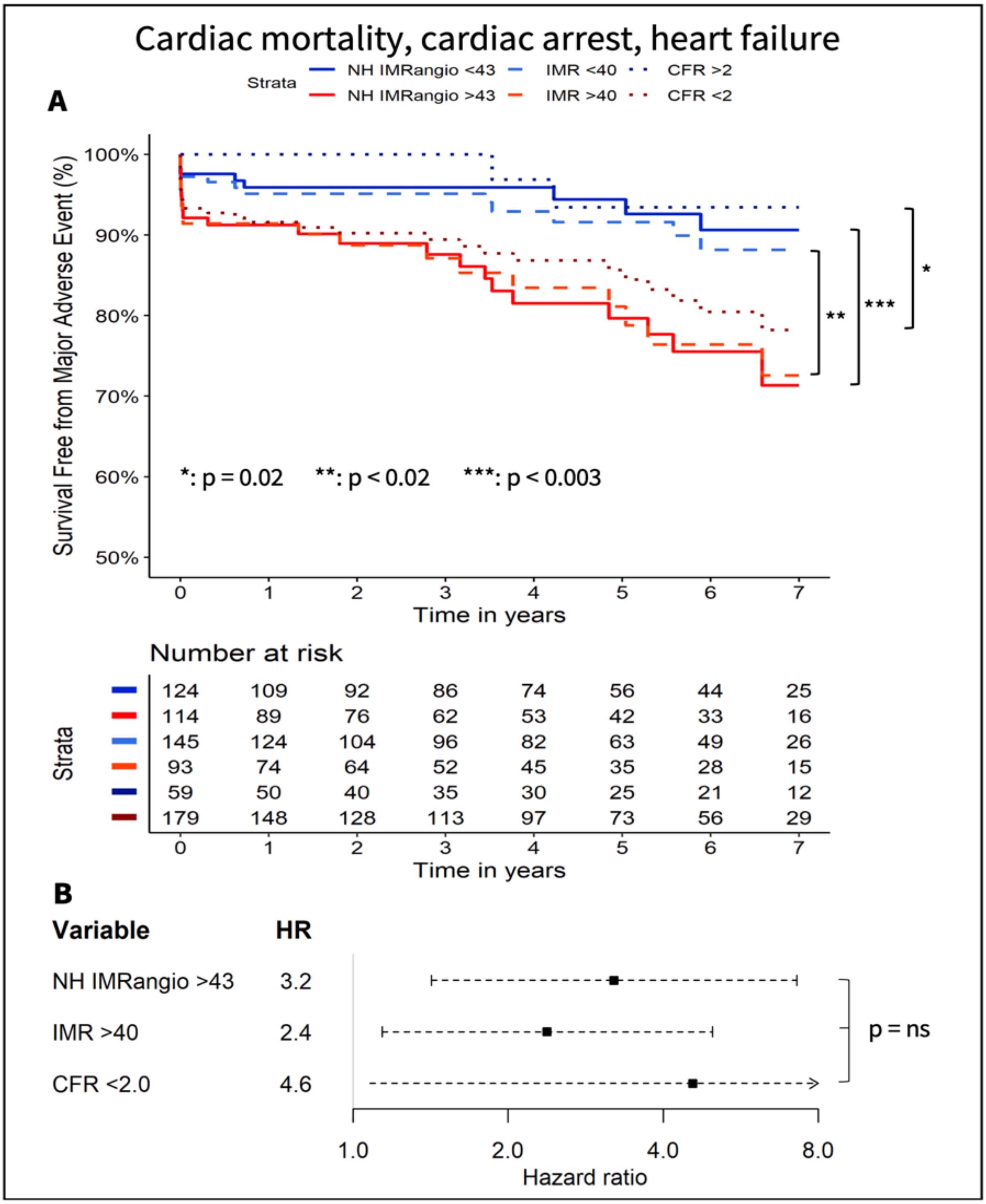

Figure S4: Kaplan Meier curves of freedom from cardiac mortality, resuscitated cardiac arrest, new heart failure diagnosis with high versus low i) NH IMR_{angio}, ii) IMR and iii) CFR **(A)**. Forrest plot displaying the hazard ratio of high i) NH IMR_{angio}, ii) IMR, and iii) CFR **(B)**. *CFR: coronary flow reserve; HR: hazard ratio; IMR: index of microcirculatory resistance; NH IMR_{angio}: non-hyperaemic IMR_{angio}; ns= not significant.*

Figure S5

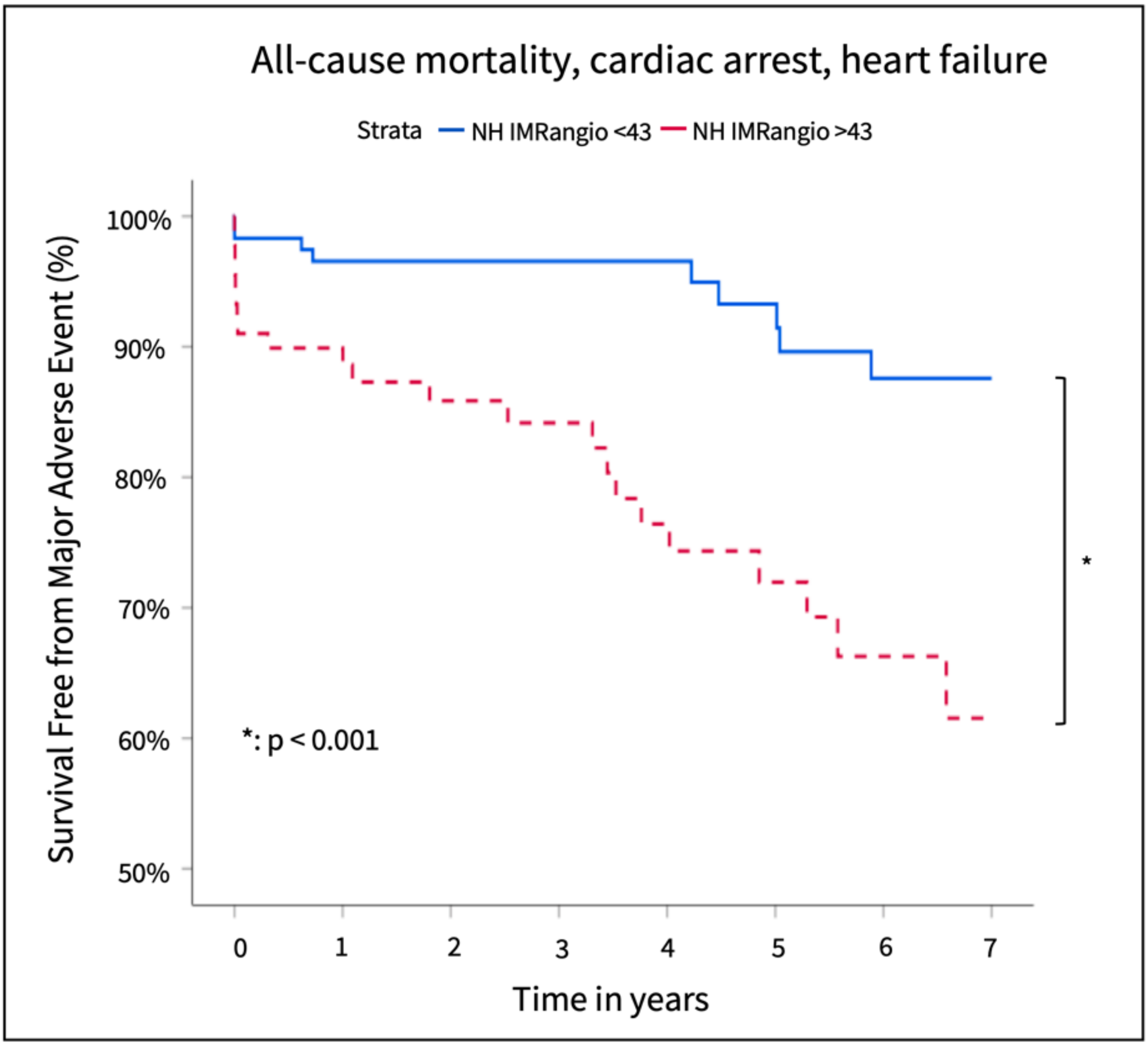

Figure S5: Kaplan Meier curves of freedom from cardiac mortality, resuscitated cardiac arrest, new heart failure diagnosis with high versus low NH IMR_{angio} in 207 patients with post pPCI TIMI Flow 3.

NH IMR_{angio}: non-hyperaemic IMR_{angio}; pPCI: primary percutaneous coronary intervention; TIMI: Thrombolysis in Myocardial Infarction.
